# Occupational Risk Evaluation through Infrared Thermography: Development and Proposal of a Rapid Screening Tool for Risk Assessment Arising from Repetitive Actions of the Upper Limbs

**DOI:** 10.3390/ijerph17103390

**Published:** 2020-05-13

**Authors:** André Luiz Soares, Antonio Augusto de Paula Xavier, Ariel Orlei Michaloski

**Affiliations:** 1 Câmpus Guarapuava, Universidade Tecnológica Federal do Paraná (UTFPR), Guarapuava, Paraná 85053-525, Brazil; 2 Câmpus Ponta Grossa, Universidade Tecnológica Federal do Paraná (UTFPR), Ponta Grossa, Paraná 84017-220, Brazil; augustox@utfpr.edu.br (A.A.d.P.X.); ariel@utfpr.edu.br (A.O.M.)

**Keywords:** risk assessment, repetitive actions, OCRA Index, infrared thermography, skin temperature

## Abstract

Risk analysis is one of the main tools for preventing the occurrence of Work-Related Musculoskeletal Disorders. New methods of risk analysis should seek to be more agile and simplified, encouraging them to be widely applied in work environments. This paper aimed to develop a rapid tool for assessing the risk of developing Work-Related Musculoskeletal Disorders (WMSDs) arising from repetitive actions of the upper limbs, while using a thermographic camera to measure skin temperature variation. A workstation was developed in an environmentally controlled laboratory, representing the five levels of risk presented by the Occupational Repetitive Actions Index (OCRA) Index, which were performed by 32 participants for 20 min. each level. There was a significant change in forearm skin temperature at all risk levels (*p* < 0.001), with a positive linear correlation (*r* = 0.658 and *p* < 0.001), which led the authors to perform linear regression analysis for the forearm region. The Predicted OCRA Index calculation equation was successfully developed (R = 0.767 and R² = 0.588), while using as independent variables: air temperature and temperature variation of the forearm skin. The Predicted OCRA Index can be applied as a screening tool for large numbers of workers in the same company or sector, due to its speed of application and the determination of risk level, but it does not replace the original OCRA Index.

## 1. Introduction

The use of new technologies and the development of new applications for existing ones are great allies to Human Factors and Ergonomics [1], not only achieving the safety and well-being of the workers, but also the prevention of injuries and performance enhancements [2,3,4,5]. The continuous work of researchers in the Human Factors and Ergonomics area seeks to improve and adapt the existing conditions, tools, and working environments, in order to provide the healthiest possible working condition to workers.

Musculoskeletal disorders (MSDs) are a one of the occupational disorders that most harm workers [6,7], occurring in different ways in several areas of the body, such as the wrist [8,9,10], hands [11,12,13,14,15,16], back [17,18], shoulders [19,20], and neck [19]. When MSDs have their cause related to work, they are called Work-Related Musculoskeletal Disorders (WMSDs) [21]; these being the most common occupational diseases in Europe [22] and, in 2018, accounted for 27.2% of occupational illnesses that occurred in private industries in the United States, with an average of 12 days away from work [23]. The prevention of WMSDs is an alternative for reducing costs and medical work leaves assigned to the workers’ health recovery, since they may lead to absence from work, causing economic and social impacts on the lives of workers and the activities of companies and their countries [24].

Methods for the risk assessment of workstations measure the probability of injuries, allowing for the managers to act preventively in the maintenance of the work environment, which in turn reduces the risks of WMSDs and may also enhance worker’s performance [4,5]. Repetitive strain injuries are one of several types of WMSDs, which can occur in the medium to long term and are slow to show symptoms, occurring mostly in the neck, shoulder, elbow, forearm, wrist, and hands, also appearing in more than one area at once [25]. 

Some of the main risk analysis methods in repetitive strain in the upper limbs are [26]: The Occupational Repetitive Actions Index (OCRA Index) [27], the Strain Index (S.I.) [28], and the Threshold Limit Value (TLV) [29]. The TLV method mainly focuses in the frequency of actions and peak force of hand activities [29,30,31]; the S.I. method is a more detailed approach that focuses on effort intensity, effort duration, efforts per minute, wrist and hand posture, work speed, and task duration per day [28,30,31]. The OCRA Index [27,30,31,32] takes numerous factors into account when diagnosing workstations: frequency of technical actions, repetitiveness, inadequate arm, elbow, wrist and hand postures, force, complementary factors (mechanical, environmental, and organizational), recovery periods, and repetitive and non-repetitive task duration. The standards EN 1005-5-Safety Of Machinery-Human Physical Performance—Part 5: Risk Assessment For Repetitive Handling At High Frequency [32] and ISO 11228-3-Ergonomics—Manual handling—Part 3: Handling of low loads at high frequency [31] and the Technical Committee on Musculoskeletal Disorders of the IEA (International Ergonomics Association) [33] recommend the utilization of the OCRA Index for assessing and rating the risks in situations of repetitive strain in the upper limbs; therefore, it was selected as the repetitive task design tool applied in this study.

The human body maintains its temperature at approximately 37 °C through several types of heat transfer, like sweat and vascular expansion [34,35,36], seeking to keep itself in a thermally comfortable situation in relation to its environment [37]. During the execution of exercises, the blood flow to the muscles used undergoes vasoconstriction and it is observed that there is an increase in skin temperature in the muscle region used during the activity performed [38,39] and that will remain during the execution of the exercise; there is also a vasodilatory response in order to facilitate heat loss through the skin, which will lead to a reduction in body temperature [34,35,39] in order to keep body temperature within acceptable limits.

A thermographic image is a graphic representation of the distribution of temperature over a target [40] with several applications in human beings, such as thermoregulation studies, breast cancer detection, diagnosis of diabetes-related vascular problems, diagnosis of bone problems, among others [12,40,41,42,43,44,45,46]. Infrared thermography (IT) is able to visualize the functions and physiological responses of the skin, allowing for observing temperature changes that are caused by alterations in the blood flow (vasodilation and vasoconstriction) [34,35], in addition to other changes, like pain [47], inflammation [48], postural alterations [49], sweating [34,35], and environmental parameters, such as air temperature, radiant temperature, air velocity, and humidity [35,50]. The several advantages of the application of IT in human beings include that it does not cause radiation-related damage [42], it is non-invasive [51], it does not require direct contact with the patient’s skin [44], and it is economically-affordable and painless [43]. The disadvantages of using IT involve the need for environmental control of variables, such as air temperature, relative humidity, and the presence of radiation sources [52,53]. In addition, the subjects analyzed must comply with guidelines prior to the experiment and they are difficult to be controlled by the researcher, such as avoiding the consumption of energy drinks, not applying skin creams or performing physical exercises [52,53,54,55]. Additionally, while performing the analysis by IT, the subject must leave the skin of the region of interest (ROI) exposed and wait for acclimatization to the environment of execution of the experiment [52].

The aim of this paper was to develop a rapid tool for assessing the risk of developing WMSDs that arise from repetitive actions of the upper limbs, using a thermographic camera to measure skin temperature variation and providing a diagnosis equivalent to the risk levels given by the OCRA Index. It is expected that the skin temperature will increase during the execution of the projected repetitive tasks, and this temperature variation will be proportional to the five risk levels proposed by the OCRA Index. To measure the skin temperature variation, 160 simulation experiments were performed in a workstation that was located in an environmentally controlled laboratory, and the skin temperature of the shoulders, forearm, and hand of the right arm were measured using IT before the repetitive task started and after 20 min. of execution of the projected repetitive task.

The Methodi Ordinatio [56] research methodology was applied in 2018 to verify whether previous works sought to apply IT for assessing the risk of developing WMSDs, which takes into account the impact factor, year of publication, and number of citations to rank the relevance of scientific articles. The following databases were used to search for articles: Science Direct, Scopus, Web of Science, Springer, and Scielo. The time interval was from 1998 (year of the first publication of the OCRA Index) to 2018, and the keywords “Infrared Thermography” and “Thermography” were searched in pairs with the keywords “OCRA”, “Work Related Musculoskeletal Disorder”, “Repetitive Strain Injury”, “Upper-Limb Musculoskeletal Disorder”, “Musculoskeletal Disorder”, and “Repetitive Movements”. The result of keyword searches in the databases led to 379 articles, of which, after, applying the steps of Methodi Ordinatio, 61 articles remained, ranked by their scientific relevance. After analyzing the results, it was found that the application of IT to assess the risk of developing WMSDs is an unprecedented application in the field of Human Factors and Ergonomics, so that the present study was carried out to bridge this gap.

## 2. Materials and Methods 

### 2.1. Ethics

The Committee of Ethics in Research of the Federal University of Technology, Paraná-Brazil analyzed this study and it received the authorization to be conducted in October 2018, under the no. CAAE 97755518.6.0000.5547, in accordance with the Declaration of Helsinki. The experiments that were conducted in this study took place in the Laboratory of Thermography at the UTFPR. 

### 2.2. Participants

The research participants were members of the academic community at the Federal University of Technology, Paraná-Brazil, randomly invited to take part. A total of 32 people volunteered to participate in the study, 16 men and 16 women, all of whom were right-handed.

Before performing the task proposed, the participants were instructed to not consume coffee, teas, energy drinks, or alcohol, not perform any type of physical activity, not smoke, not apply creams or oils on the skin of their upper limbs, not receive any type of therapy (cryotherapy, electrotherapy, ultrasound, heat treatments, massages, acupuncture, hydrotherapy, etc.), not expose themselves to the sun or rain, not have a history of MSDs or WMSDs in the upper limbs, and to not participate in the experiments during the menstrual period [52,53]. The previously mentioned factors were applied as a criterion for the exclusion of participants in the study, as they are actions/conditions that could influence the skin temperature of the participant during the execution of the experiments proposed in this work, according to literature [52,53].

Table 1 presents the participant’s description, showing the mean and standard deviation of age, weight, height, and Body Mass Index (BMI).

The OCRA Index does not differentiate the gender of the worker assessed when calculating the risk level of a repetitive task. Therefore, in this study, the same number of male and female participants was maintained and the data analysis will always be performed for the entire set of participants.

### 2.3. Workstation

**Figure 1:**
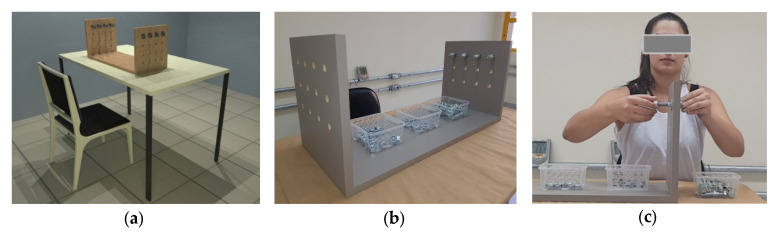
Project of the workstation employed in the study, involving the assembly and disassembly of screws, nuts and ferrules: (**a**) Workstation design; (**b**) Built workstation; and, (**c**) Female participant performing the designed repetitive task.

The application of IT in human studies involves the need to control environmental variables [40], so a workstation Figure 1a–c was designed in a laboratory, involving the assembly and disassembly of screws, nuts, and ferrules, at a sitting position, being inspired by the work of Govindu and Babski-Reeves [57].

The OCRA Index ranks repetitive tasks according to three degrees of risk: Acceptable (green), Conditionally Acceptable (yellow), and Not Acceptable (red). However, international standards [31,32] classify the “Not Acceptable” condition into three further degrees of risk: Low Risk (orange), Medium Risk (red), and High Risk (purple). Table 2 presents the risk degrees employed in this study:

The calculation of the OCRA Index involves the following factors: frequency of technical actions and repetitiveness, inadequate arm, elbow, wrist and hand postures, force, complementary factors (mechanical, environmental, and organizational), recovery periods, and repetitive and non-repetitive task duration. The design of the task applied in this study simulated five degrees of risk, which are presented in Table 3 and Table 4. Complementary factors were not applied in the task design of this study.

The calculation of the OCRA Index represents a complete work shift, in this case projected for a 9 h journey, but the analysis of individual repetitive task cycles alone is sufficient for the calculation of the entire shift index as long as only one task type is performed throughout the work shift [27,31,32], which is the case of this study. The participants should perform the task for 20 min. or until exhausted according to the parameters presented in Table 3 and Table 4. The time of 20 min. was selected because during the execution of physical exercises the human body reaches its heat balance between 30 to 45 min. of exercise; however, the temperature increase occurs between 15 to 20 min. of physical activity [58].

A metronome was set to mark the moment of performing each technical action in the task of assembling and disassembling screws, nuts, and ferrules in order to aid the participant in meeting the work pace established in Table 3. However, the OCRA Index was recalculated according to the performance of each person due to the individual circumstances of dexterity and speed. Each participant was filmed performing the task, so that it was possible to analyze the posture and rhythm adopted by each participant. Each participant was filmed performing repetitive task cycles, so that it was possible to analyze the posture and rhythm adopted by everyone. The obtained videos were then analyzed in slow motion using Kinovea™ software. Video editing software is required, as each technical action must be timed and inserted in the OCRA Index calculation. The software used also allows for the angle of upper limb postures to be determined according to the “Posture” item presented in Table 4. All of the participants were able to perform the tasks at the proposed pace and posture, and there was no dropout before the proposed 20 min. repetitive task period.

All 32 participants performed the five levels of repetitive effort proposed in Table 3 and Table 4, leading to 160 results of skin temperature variation for each region of interest analyzed (as described in Section 2.5).

### 2.4. Equipment

The environmental variables air temperature and air relative humidity were measured by two thermo hygrometers ICEL HT-208™ (ICEL, Itabira, Brazil) that were placed around the participant performing the task, at a 1-m height. At least one digital display must indicate the ambient temperature at all times in the laboratory where the thermographic experiments are performed, as the temperature must be maintained between 18 °C and 25 °C and held for at least one hour to better than 1° [52,53]; relative humidity should be maintained between 40% and 70% [53]. The average air temperature and relative humidity were, respectively, 20.99 ± 1.63 °C and 54.09 ± 7.85% during the experiments.

A thermographic camera model FLIR T440™ (FLIR Systems, Tallinn, Estonia), placed on a tripod 2.8 m away from the participants and at a 1-m height, measured the skin temperature. The distance and height of the thermographic camera should allow for observing the entire region of interest (ROI), according to the researcher’s objective [52,53]. However, the distance must be kept constant for all patients if the objective of the study is to compare subjects, since the number of pixels in the ROI can provide different temperature readings if the distance varies from one subject to another [59]. The software FLIR Tools™ (FLIR Systems, Tallinn, Estonia) was employed to analyze the thermal images, because it allows ROIs to be selected in a variety of formats, providing temperature readings of the hottest point, coldest point, and the average temperature of the region of interest.

### 2.5. Infrared Thermography (IT): Experimental Procedures

The IT obtains the temperature of a target surface by measuring the radiation emitted by the body, provided that its emissivity is known [53]. A value of 0.98 for emissivity of skin was adopted for all of the participants [60]. 

**Figure 2:**
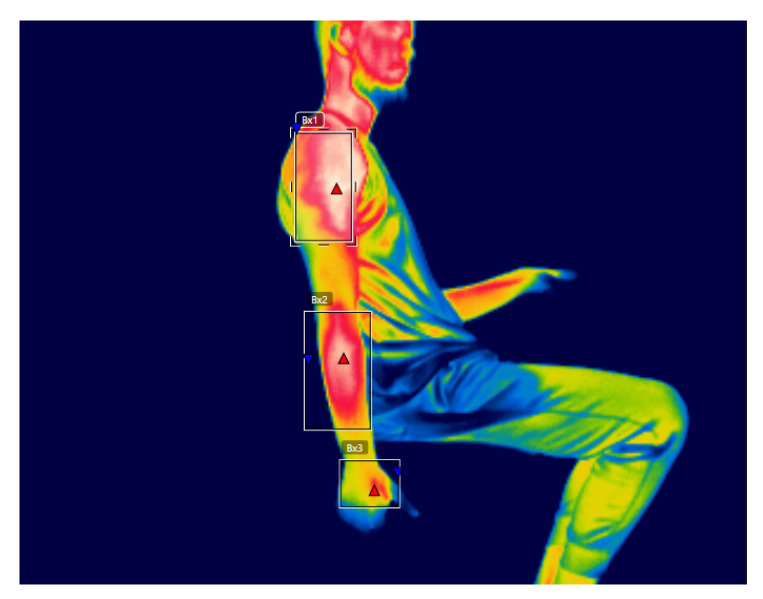
Indication of the regions of interest analyzed, shown in a male participant, after performing one of the experiments of the study for 20 min.

This study had the purpose of examining the right upper limb, dividing it into three ROIs Figure 2 [54]: shoulder, forearm, and hand. Those ROIs were selected, because they correspond to the upper limb ROIs employed to describe the posture of in the OCRA Index [27], as shown in Figure 2. The hottest point in each ROI was selected, rather than the average between the hottest and coldest point, to avoid errors that are caused by misselecting a cold point in the background of the image [53,61]. The software FLIR Tools™ was employed to determine the hottest point in each ROI and participant.

Each participant waited for the thermal acclimatization for 20 min., wearing the standard clothing (a white sleeveless t-shirt) provided by the study and maintaining a sitting position throughout the experiment. Thermal images were gathered at the beginning and immediately after the end of the task, with a 20 min. interval between images. Each thermal image displays all of the selected ROIs that are described in Figure 2.

### 2.6. Statistical Analysis

The following statistical tests were performed:

- paired *t*-test: to find out whether significant temperature variation occurred after the performance of the task in each OCRA level (per ROI); to compare the actual OCRA Index values and the predicted values calculated using linear regression;
- correlation test: correlation analysis was performed between the results obtained per ROI and the OCRA level performed by each participant; and,
- regression analysis: to determine equations that allow for calculating the OCRA level of each repetitive task, using the temperature variation of each ROI as input information.

The software IBM SPSS Statistics Version 24^®^ was employed to conduct all of the statistical analyses. When applicable, the results of standard deviation (SD), standard error (SE), degrees of freedom (dF), and significance (*p*-value) will be presented.

## 3. Results

In this section, the obtained results will be presented through descriptive statistics. Complete data are available in Supplementary Materials.

### 3.1. OCRA Index

Table 5 presents the results of the calculated OCRA Index for each risk level. The mean, standard deviation, standard error, minimum, and maximum results are shown according to the risk level of the OCRA Index.

According to Table 2, all of the results shown in Table 5 are in accordance with the risk levels that were proposed by the OCRA Index. This means that the workstation was correctly designed and the repetitive tasks planned in Table 3 and Table 4 allowed for correctly simulating the desired repetitive efforts. However, specifically regarding the “Conditionally Acceptable” risk level (Yellow), it is observed that 17 participants failed to stay within the range that is shown in Table 2. It is observed that 16 participants did not reach the proposed minimum effort level for the risk level “Conditionally Acceptable”, falling below 2.2, while one participant performed a risk level of 3.92, rated “Low Risk”.

### 3.2. Variation of Skin Temperature

**Figure 3:**
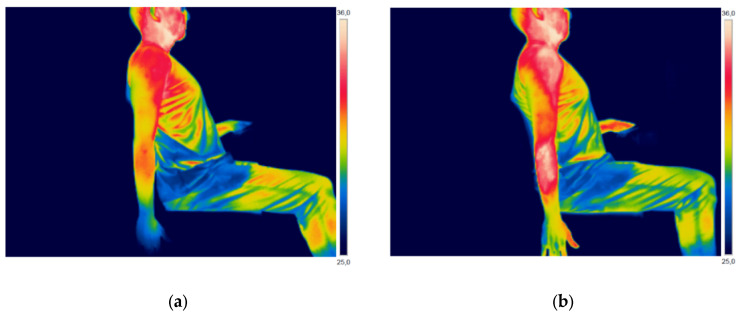
Thermographic image of a male participant: (**a**) before performing the high-risk repetitive task; and, (**b**) after performing the high-risk repetitive task.

**Figure 4:**
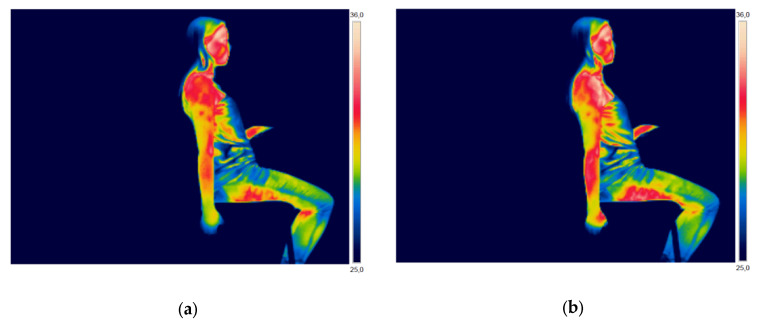
Thermographic image of a female participant: (**a**) before performing the high-risk repetitive task; and, (**b**) after performing the high-risk repetitive task.

The IT was employed to verify and quantify the variation of skin temperature at the shoulder, forearm, and hand. Figure 3 (man) and Figure 4 (woman) present thermographic images of two participants, before and after performing the high-risk repetitive task that was designed for this study (Purple Zone).

Figure 3 and Figure 4, through visual analysis, show an apparent increase in skin temperature in the three ROIs analyzed in this study: shoulder, forearm, and hand. However, visual analysis is not enough to quantify and analyze the variation in skin temperature, so the thermal images were analyzed using software.

Table 6 presents the results for the variation of shoulder skin temperature according to the risk level. 

The mean variation in shoulder skin temperature was positive at all risk levels that were performed by participants. There is also a gradual increase in temperature variation according to the risk level, with a mean increase of 0.881 °C in the “Acceptable” risk level (green) and 1.772 °C in the “High” risk level (purple). At the green risk level, a single participant had a final temperature that was lower than the initial temperature after the experiment was performed.

Table 7 presents the results for the variation of forearm skin temperature according to risk level. 

The mean variation in forearm skin temperature was positive at all risk levels that were performed by participants. There is also a gradual increase in temperature variation according to the risk level, with a mean increase of 0.706 °C in the “Acceptable” risk level (green) and 2.488 °C in the “High” risk level (purple). The minimum temperatures show that some participants showed a reduction in the skin temperature after performing 20 min. of repetitive effort in the green and yellow zones; however, the descriptive statistics show that the average of the participants showed an increase in the skin temperature at these same risk levels.

Table 8 presents the results for the variation of hand skin temperature according to risk level. 

When analyzing the ROI of the hand, the “Acceptable” (green) and “Conditionally Acceptable” (yellow) risk levels showed mean variation indicating temperature reduction, while the “Low” (orange), “Medium” (red), and “High” (purple) risk levels showed a temperature increase. The skin of the hand also showed the highest standard deviation values, indicating greater variability in results.

The difference between the analyzed ROIs is noticeable when analyzing the temperature variation through the paired t-test. In the shoulder and forearm, a significant temperature difference (*p* < 0.001) occurred at all risk levels after 20 min. of repetitive task execution, even at the “Acceptable” risk level (green). The temperature of the hand skin showed no significant temperature difference after the experiment in the levels “Acceptable” (green) and “Conditionally Acceptable” (yellow), but, at the highest risk levels, it was possible to observe significant differences in skin temperature (*p* < 0.005) after performing repetitive work for 20 min.

### 3.3. Correlation Analysis

Correlation analysis measures the degree of association between two variables, seeking to determine whether there is a linear relationship between them [62]. Table 9 shows the correlation coefficients and significance for the correlation tests between temperature variation (ΔT) and the risk zone of the OCRA Index: ΔT_S × OCRA for the shoulder, ΔT_F × OCRA for the forearm, and ΔT_H × OCRA for the hand.

According to the results that are presented in Table 10, all the ROIs analyzed showed positive and significant linear correlations (*p* < 0.001) when correlated with the ROI skin temperature variation and the level of risk that the participant performed in his repetitive task, but with different intensities. 

The forearm showed the best correlation with *r* = 0.658, while the ROIs for the shoulder and hand showed Pearson correlations very similar and smaller than the forearm region. Therefore, the proposal of this article will only be developed for the forearm region, since it presented the strongest correlation with the repetitive effort performed by the participants.

### 3.4. Regression Analysis

One of the objectives of applying regression analysis is to predict a phenomenon based on the measurement of one or more variables. The aim of this study was to formulate an equation that would allow the OCRA Index to be calculated by measuring the variation in skin temperature of one of the three ROIs analyzed in this laboratory study. Table 10 presents the results that were found for the regression analysis for the forearm skin.

Based on the results that are presented in Table 10, the following equation for calculating the OCRA Index is formulated as a function of the variation of forearm skin temperature:

OCRA = 1.684 + (2.181.ΔT_F),(1)

**Figure 5:**
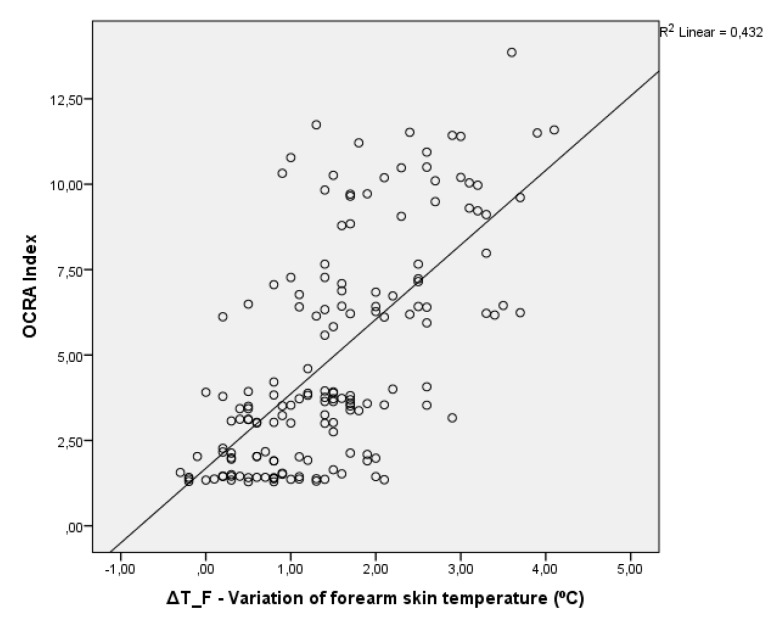
OCRA Index x ΔT_F–Variation of forearm skin temperature.

Equation (1) has a correlation of *R* = 0.658 and *R²* = 0.432. This means that forearm skin temperature variation explains 43.2% of the variation in the OCRA Index according to the results of this study. Figure 5 presents the scatter plot and plot of Equation (1) for the linear regression performed.

However, other personal and environmental variables were measured during this study. A multiple regression analysis was also performed using the following variables in order to check whether it was possible to obtain better predictions of the OCRA Index using other variables: gender (male and female), age (years), weight (Kg), height (m), BMI (Kg/m²), air temperature (°C), relative humidity (%), and variation of forearm skin temperature (°C). Table 11 shows the results obtained after the multiple regression analysis.

The multiple regression analysis using all of the variables presented in Table 11 showed *r* = 0.746 and R² = 0.557, showing greater predictability than Equation (1). However, it is observed that only the air temperature and the skin temperature variation of the forearm showed significant correlation with the proposed model (*p* < 0.001). In addition, the residual analysis indicated the presence of five outlier results that could influence the correlation and regression indexes. Therefore, a last multiple regression analysis was performed in Table 12, after excluding outliers and using independent variables: air temperature and forearm skin temperature variation.

Based on the results that are presented in Table 12, the following equation for calculating the OCRA Index is formulated using as independent variables: air temperature (Ta) and forearm skin temperature variation (ΔT_F):

OCRA = −10.173 + (0.567Ta) + (2.083ΔT_F)(2)

**Figure 6:**
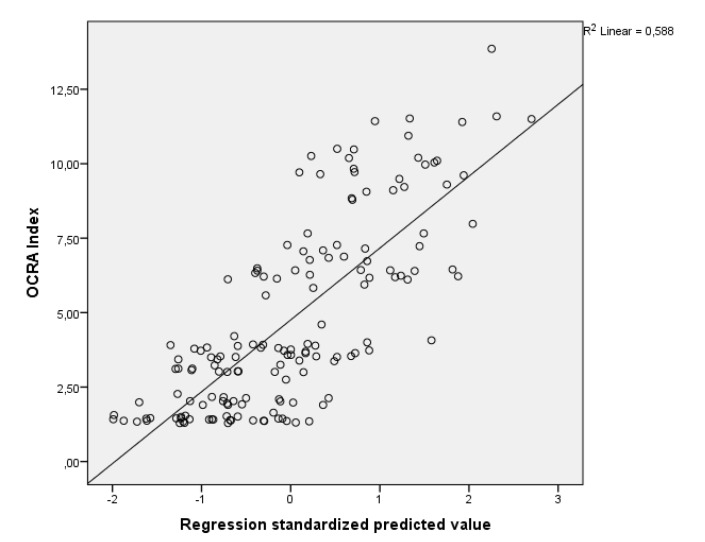
OCRA Index x Standardized predicted value, according to Equation (2).

Equation (2) has a correlation of R = 0.767 and R² = 0.588. This means that air temperature and forearm skin temperature variation explain 58.8% of the variation in the OCRA Index according to the results of this study and it has significant correlation with the model. Figure 6 presents the scatter plot and the plot of Equation (2) for the multiple regression performed.

The use of air temperature and forearm skin temperature variation as independent variables provided better results in relation to the prediction of the OCRA Index and the correlation of the independent variables. The paired t-test was applied in Table 13 by comparing the calculated results of the OCRA Index (summarized in Table 6) and the predicted results calculated using Equation (2). The *p*-value of 1.000 shows that the results found using Equation (2) are statistically equal (*p* > 0.05).

However, despite the results obtained showing that the calculated OCRA Index and Predicted OCRA Index are statistically equal, it should be noted that the correlation between the two methods was not perfect (R = 0.767), which is, there are differences between the results obtained by each method.

### 3.5. Proposal of a Rapid Tool for Risk Assessment Through Infrared Thermography Arising from Repetitive Actions of the Upper Limbs

**Figure 7:**
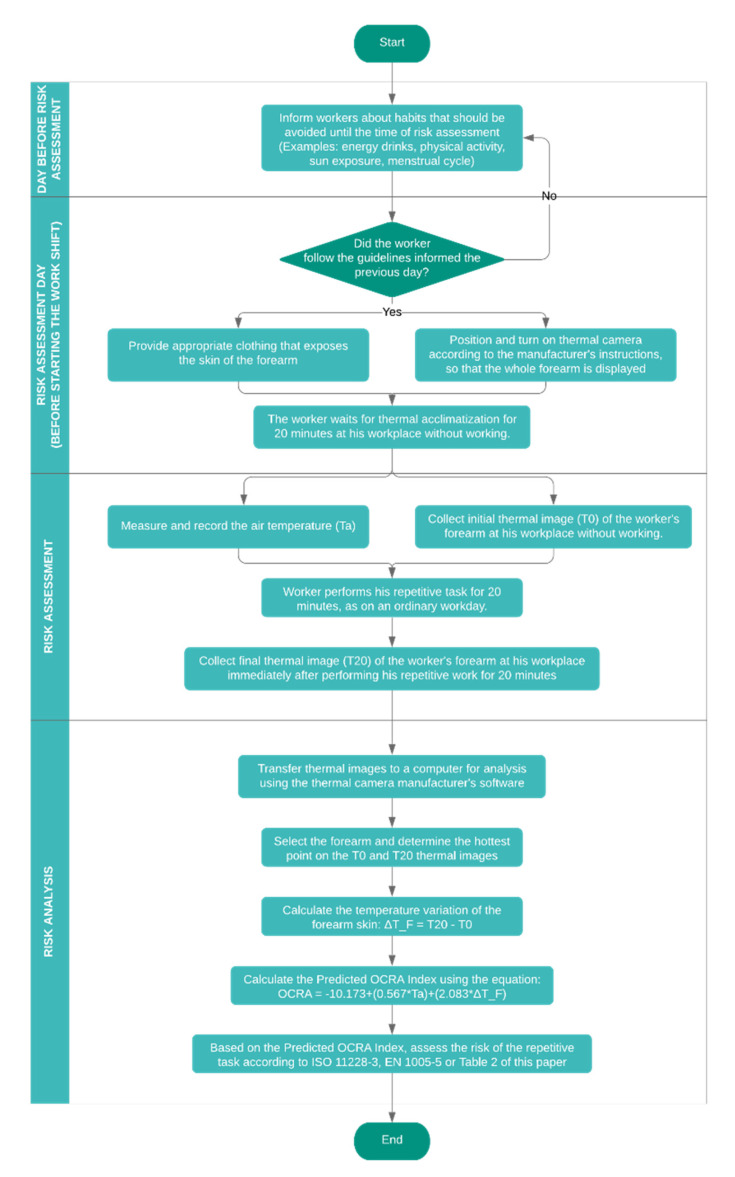
Proposal of a rapid tool for risk assessment through infrared thermography arising from repetitive actions of the upper limbs.

The rapid tool for risk assessment through infrared thermography arising from repetitive actions of the upper limbs is proposed in Figure 7 after demonstrating the development of this tool in the previous sections.

The proposal for this study has four phases of application:

- Phase 1—Day before risk assessment: instructions that must be passed on to workers who will participate in the risk assessment the day before the analysis, as they may interfere with the results. Examples: energy drinks, physical activity, sun exposure, andd menstrual cycle;
- Phase 2—Risk assessment day (before starting the work shift): actions that must be taken before the risk assessment begins, by the risk evaluator and the worker;
- Phase 3—Risk assessment: execution of the repetitive task by the worker and application of IT by the risk evaluator;
- Phase 4—Risk analysis: transfer of thermal images and computer analysis, application of equation 2 to calculate the Predicted OCRA Index and final risk diagnosis;

## 4. Discussion

The aim of this study was to develop a rapid tool for assessing the risk of developing WMSDs arising from repetitive actions of the upper limbs, while using skin temperature variation as an independent variable. Monitoring skin temperature might provide important information about the performance and reaction of muscles and joints [48,63], which in turn might indicate the presence or development of musculoskeletal disorders [18,63,64]. The study of the skin temperature of individuals during sports, exercises, and office tasks has been performed by authors prior to the execution of this study [10,14,16,20,47,57]. However, the study of skin temperature behavior in relation to the various risk levels proposed by the OCRA Index makes this study unprecedented, as shown in the Introduction.

The results found demonstrated an increase in the skin temperature and a significant difference between the initial and final temperature in all levels of repetitive effort performed when analyzing the forearm and shoulder (*p* < 0.001). Other works also conducted temperature analysis of upper limbs, like shoulder, arm, and forearm, presenting a temperature increase in the ROIs examined after the performance of an effort or labor [10,14,16,20,47,57]. The reduction in hand skin temperature that was found in this study might be due to the low level of repetitive effort present in lower risk tasks, where fewer technical actions occur at lower movement frequencies, as shown in Table 3 and Table 4, and mainly, for the vasoconstrictor response of the skin that occurs in response to physical exercises [53,65]. Skin temperature reduction during exercise has been found by other studies [66], but especially in office tasks whose repetitive stress level is not high (such as typing and mouse use), there have been reductions in the temperature of the hand skin, as in the Gold et al. [14] study. The prolonged use of objects that require manual strength might also lead to a decrease in hand temperature, as seen in the use of knives [13] and, in this study, the hands were in contact with metallic objects during part of the work cycle.

When analyzed from the perspective of the correlation analysis, it was observed that the forearm region presented the greatest correlation between the variation of the skin temperature and the repetitive task performed, which led the authors to choose this ROI for the development of the proposed tool. The choice of the forearm for application of the tool also provides advantages in the practical application of the assessment tool, as a sleeveless T-shirt is not necessary to perform the risk analysis, since it is common for the skin of the forearm to be exposed. The graph presented in Figure 5 shows that there is a linear correlation between the OCRA Index and the variation in the temperature of the forearm skin. However, it should be noted that, as the intensity of the task increased, the dispersion of data also increased, while, at lighter levels of repetitive task, a greater correlation occurred. This also indicates that the lighter tasks, with lower speed and skill and therefore easier to be performed by the participants, obtained more stable correlation results; the more complex tasks demanded greater dexterity and speed from the participants, which led to results with greater variability and mostly concentrated on the left side of the regression line. This variability could lead to an underestimated risk diagnosis, so the application of the tool proposed in this study should be performed with caution.

The initial objective of this paper was to only use the variation in skin temperature as an independent variable, as this would make risk analysis even simpler. However, when multiple regression was performed, it was noticed that the air temperature also has a significant influence with the model (*r* = 0.422 and *p* < 0.001) that should not be ignored. Adding the air temperature made the equation of the Predicted OCRA Index more robust, raising the model’s *r* value to 0.767 and R² = 0.588. Air temperature has an influence on skin temperature, especially when fully exposed to the environment [67], and the influence of air temperature on skin temperature has already been reported by other studies [53,68]. It was also noted through the results obtained in Table 11 that the demographic factors of gender, age, weight, height, and BMI did not present a significant correlation with the variation of skin temperature, and, therefore, were not included in Equation (2). Such results are in agreement with the original OCRA Index [31,32], since it also does not take into account demographic factors, which makes its application simpler, both for the OCRA Index and the Predicted OCRA Index. However, even if the regression equation presented Equation (2) does not include the demographic factors analyzed, the existing literature demonstrates that it is possible that they have an influence on skin temperature. Men and women are proven to have differences in metabolism, subcutaneous fat, in addition to the menstrual cycle [53]. In this study, measures were taken (as described in the Section 2.2—Participants) in order to reduce the influence of factors other than just the task performed. According to the obtained results, the value of *r* = 0.767 demonstrates that there are other factors that influence the skin temperature that were not covered by this study, which is therefore a limitation of this study.

The strongest point of the assessment tool that was proposed in this study, the Predicted OCRA Index, is the application of IT through thermal cameras. The use of IT in studies with human beings has several advantages already mentioned in the Introduction, such as not needing contact with the patient, being painless, and non-invasive. The use of thermal cameras also has the advantage of being small and easy to transport. Equipment portability is essential, seeing that the accurate assessment of a repetitive strain via the OCRA Index [27] requires an in loco visit to the workplace for a detailed observation of the work journey. This portability allows the worker to remain at their workstation during the assessment. The second strongest point of using the Predicted OCRA Index is the reduction of data that needs to be collected to perform the risk assessment of development of WMSDs, since only two variables are necessary: air temperature and forearm skin temperature variation. The traditional application of the OCRA Index involves collecting a much larger amount of data [27,31,32]: frequency of technical actions, repetitiveness, inadequate arm, elbow, wrist and hand postures, force, complementary factors (mechanical, environmental, and organizational), recovery periods, and repetitive and non-repetitive task duration. The third strong point is the speed of application of the Predicted OCRA Index, with this advantage being a direct consequence of the reduction in the number of variables that must be collected in order to calculate the OCRA Index and the level of risk of developing WMSDs.

The weakest point in using the Predicted OCRA Index comes from one of its strengths: the number of variables collected for risk assessment. Although the OCRA Index has a more complex and slow application, the amount of details provided along with the risk diagnosis allows for the evaluator to design improvement actions focused on the variables that present the worst performance, allowing corrective measures to be implemented and improve working conditions [31,32]. According to the study by Antonucci [30], when comparing the OCRA Index with other risk analysis tools (Strain Index and ACGIH), the OCRA Index proved to be the most complete method due to the amount of details provided during the risk assessment. Another weakness is the lack of application of the Predicted OCRA Index in real work environments. 

Some limitations of this work need to be highlighted. The sample size is only 32 participants (16 men and 16 women), which does not guarantee that the results that were found in this study will be the same as for larger samples. In addition, the participants were healthy and mostly young members of the academic community, and they do not necessarily represent workers susceptible to suffering WMSDs. This was a choice of the authors of this study to reduce as much as possible the influence of variables other than the repetitive task performed; however, it generates limitations to the study.

Finally, the Predicted OCRA Index has the forearm skin temperature variation as its main variable, and this variable does not have as its only influence the task performed (as seen in the correlation and regression results). The thermoregulatory system of the human being is extremely complex [34,35], possessing influence of various factors beyond the task performed by humans, such as vasodilation, vasoconstriction, and sweat evaporation [69]. Additionally, the use of different clothing in real work environments, with larger or smaller areas of exposed skin might affect the thermoregulation and skin temperature. The experiments presented in this study were carried out in an environmentally controlled laboratory in relation to the air temperature, and with the standardization of the clothing used by the participants, leaving the skin of the regions of interest fully exposed. Applying IT in a real work environment will hardly have the same application conditions. It is common to have several radiation sources in work environments, in addition to unstable temperature conditions [67]. The previously cited studies provide extensive information regarding the influencing factors when carrying out studies using IT in humans. Briefly, Fernández-Cuevas et al. [53] divides these factors into: environmental factors (room size, ambient temperature, relative humidity, etc.), technical factors (camera features, software, statistical analysis, etc.) and individual factors (metabolic rate, genetic, gender, skin emissivity, etc.). For these reasons, the authors recommend that the works of Fernández-Cuevas et al. [53] and of Ring and Ammer [52] should be applied when interventions in real work environments are performed while using IT and the method proposed in this paper. This means that when applying the Predicted OCRA Index, all of the steps proposed in Figure 7 must be applied, in addition to that the analyzes must be performed indoors, away from radiation sources, air temperature must be maintained between 18 °C and 25 °C and held for at least one hour to better than 1 °C [52,53], and relative humidity should be maintained between 40% and 70% [53]. The application of the Predicted OCRA Index in conditions different from those performed in this study may present different results regarding the variation of the skin temperature and correlation with the repetitive task performed.

## 5. Conclusions

When comparing the strengths and weaknesses presented, as well as the results that were obtained in the process of developing the proposed method, the authors conclude that the Predicted OCRA Index does not have the potential and precision to replace the original OCRA Index, nor was this its objective. The limitations of the study, as well as the results found, suggest that the Predicted OCRA Index should not be used as a tool for analyzing risks arising from repetitive movements of the upper limbs; however, the Predicted OCRA Index can be applied as a screening tool for large numbers of workers in the same company or sector, due to its speed of application and the determination of risk level. This research recommends that, when IT technology is available, the Predicted OCRA Index is applied for screening the risk level of jobs, and for jobs that are at high risk after analysis by IT, it is recommended the application of the original OCRA Index to explore the factors that must be corrected to reduce the risk of developing WMSDs.

The authors chose the forearm region to develop the method based on the correlation results obtained Table 9, but all of the regions analyzed in the study (shoulder, forearm, and hand) showed a significant correlation with the repetitive task performed. The forearm region was selected because the objective of the study was to create a simple and quick application tool, so only the region of interest that showed the greatest correlation with the task performed by the participants was used. It is possible that, if all the ROIs analyzed in the study were used, a regression equation with higher values of R and R² would be obtained; however, this would make the application of the method more complex, therefore escaping the objective of the study.

Making risk assessment in workstations a more agile task using IT was the study proposal, because it is believed that the more workstations are evaluated, the greater the opportunities for improvement and injury prevention. The reduction in the occurrence of WMSDs is a consequence that might occur as prevention measures are implemented. The authors recommend and expect as future studies that the Predicted OCRA Index tool will be applied in the most diverse jobs and that its results be presented to the scientific community.

## Figures and Tables

**Table 1:** Participants’ characteristics.

Characteristic	Total (*n* = 32)	Male (*n* = 16)	Female (*n* = 16)
Age (years)	25.84 (4.90)	26.38 (5.25)	25.31 (4.63)
Weight (kg)	71.16 (15.00)	77.63 (15.60)	64.69 (11.52)
Height (m)	1.72 (0.10)	1.78 (0.08)	1.65 (0.07)
BMI (Kg/m²)	24.05 (4.00)	24.29 (3.99)	23.82 (4.14)

**Table 2:** Risk levels defined by the Occupational Repetitive Actions Index (OCRA) Index.

Zone	OCRA Index	Risk Level
Green	OCRA ≤ 2.2	Acceptable
Yellow	2.2 < OCRA ≤ 3.5	Conditionally Acceptable
Orange	3.5 < OCRA ≤ 4.5	Low
Red	4.5 < OCRA ≤ 9	Medium
Purple	OCRA > 9	High

**Table 3:** Repetitive task parameters designed for this study according to OCRA Index risk levels: Recovery Periods, Technical Actions, and Repetitiveness.

Zone	Recovery Periods	Technical Actions and Repetitiveness
Green	Journey: 9 h; Break for meal: 1 h break; Other breaks: 20 min. Non-repetitive tasks: 20 min; Experiment: 20 min.	Rate of 40 bpm per technical action, 19 technical actions, 40-s cycles and 660 cycles per journey.
Yellow		Rate of 50 bpm per technical action, 24 technical actions, 38-s cycles and 694 cycles per journey.
Orange		Rate of 60 bpm per technical action, 23 technical actions, 32-s cycles and 825 cycles per journey.
Red		Rate of 60 bpm per technical action, 41 technical actions, 42-s cycles and 623 cycles per journey.
Purple		Rate of 110 bpm per technical action, 51 technical actions, 42-s cycles and 630 cycles per journey.

**Table 4:** Repetitive task parameters designed for this study according to OCRA Index risk levels: Posture and Force.

Zone	Posture	Force
Green	Arm: abduction < 45°; Elbow: pronation, supination, flexion and extension > 60°; Wrist: ulnar deviation > 20°; Hand: pinch grip.	Actions that required lifting pieces corresponded to 0.5 in the Borg Scale, while other cases corresponded to 0.
Yellow		
Orange	Arm: abduction between 45° and 80°; Elbow: pronation, supination, flexion and extension > 60°; Wrist: ulnar deviation > 20°; Hand: pinch grip.	
Red		
Purple		

**Table 5:** Calculated OCRA Index.

Zone	Mean	Standard Deviation	Standard Error	Minimum	Maximum
Green	1.413	0.082	0.014	1.29	1.64
Yellow	2.563	0.606	0.107	1.90	3.92
Orange	3.716	0.291	0.051	3.16	4.60
Red	6.562	0.519	0.092	5.58	7.66
Purple	10.261	1.149	0.203	7.98	13.86

**Table 6:** Variation of shoulder skin temperature (ºC).

Zone	Mean	Standard Deviation	Standard Error	Minimum	Maximum	Paired *t*-Test	
						dF	*p*-Value
Green	0.881	0.621	0.120	−0.60	1.80	31	0.000 *
Yellow	1.175	0.594	0.105	0.00	2.50	31	0.000 *
Orange	1.425	0.655	0.116	−0.40	2.80	31	0.000 *
Red	1.734	0.691	0.122	0.30	2.90	31	0.000 *
Purple	1.772	0.666	0.118	0.60	3.10	31	0.000 *

* Significant difference.

**Table 7:** Variation of forearm skin temperature (°C).

Zone	Mean	Standard Deviation	Standard Error	Minimum	Maximum	Paired *t*-Test	
						dF	*p*-Value
Green	0.706	0.629	0.111	−0.30	2.10	31	0.000 *
Yellow	0.869	0.594	0.105	−0.10	2.00	31	0.000 *
Orange	1.400	0.663	0.117	0.00	2.90	31	0.000 *
Red	1.919	0.858	0.152	0.20	3.70	31	0.000 *
Purple	2.488	0.865	0.153	0.90	4.10	31	0.000 *

* Significant difference.

**Table 8:** Variation of hand skin temperature (°C).

Zone	Mean	Standard Deviation	Standard Error	Minimum	Maximum	Paired *t*-Test	
						dF	*p*-Value
Green	−0.150	1.080	0.191	−2.90	2.00	31	0.438
Yellow	−0.022	0.945	0.167	−1.70	2.30	31	0.897
Orange	0.344	0.950	0.168	−1.10	2.40	31	0.049 *
Red	0.581	1.126	0.199	−1.10	2.80	31	0.006 *
Purple	1.150	1.256	0.222	−1.40	3.70	31	0.000 *

* Significant difference.

**Table 9:** Correlation Coefficient and Significance of ΔT x OCRA per region of interest (ROI).

Statistics	ΔT_S × OCRA	ΔT_F × OCRA	ΔT_H × OCRA
*N*	160	160	160
Pearson Correlation (*r*)	0.426	0.658	0.423
*p*-value	0.000 *	0.000 *	0.000 *

* Significant correlation.

**Table 10:** Regression analysis using as independent variable: forearm skin temperature variation (ΔT_F).

Model	B	95% B Confidence Interval	Standard Error	*t*	Pearson Correlation (*r*)	Significance
Constant	1.684	0.989–2.379	0.352	4.786	-	0.000
ΔT_F (°C)	2.181	1.788–2.573	0.199	10.973	0.658	0.000 *

* Significant correlation.

**Table 11:** Regression analysis using as independent variables: gender, age, weight, height, BMI, air temperature, relative humidity, and forearm skin temperature variation.

Model	B	Pearson Correlation (*r*)	Standard Error	*t*	Significance
Gender (M; F)	0.893	0.017	0.503	1.775	0.078
Age (years)	−0.035	0.038	0.041	−0.852	0.395
Weight (Kg)	0.174	0.002	0.110	1.587	0.115
Height (m)	−14.045	−0.020	9.596	−1.464	0.145
BMI (Kg/m²)	−0.480	0.019	0.329	−1.462	0.146
Air temperature (°C)	0.614	0.439	0.115	5.325	0.000 *
Relative humidity (%)	−0.010	−0.090	0.023	−0.422	0.673
ΔT_F (°C)	2.116	0.658	0.193	10.983	0.000 *

* Significant correlation.

**Table 12:** Regression analysis using as independent variables: air temperature (°C) and forearm skin temperature variation (°C).

Model	B	95% B Confidence Interval	Standard Error	*t*	Pearson Correlation (*r*)	Significance
Constant	−10.173	−14.406–5.939	2.143	−4.747	-	0.000
Air temperature	0.567	0.362−0.772	0.104	5.458	0.422	0.000 *
ΔT_F	2.083	1.749−2.418	0.169	12.305	0.713	0.000 *

* Significant correlation.

**Table 13:** Paired t-test of related samples comparing the calculated results of the OCRA Index and the predicted results calculated using Equation (2).

Paired *t*-Test	Standard Deviation	*N*	dF	*t*	*p*-Value
Calculated OCRA Index x Predicted OCRA Index	2018	155	154	0.000	1000

## Supplementary Materials

The complete data of this study are available online at https://www.mdpi.com/1660-4601/17/10/3390/s1.
