# Supplementary material for: Occupational Risk Evaluation through Infrared Thermography: Development and Proposal of a Rapid Screening Tool for Risk Assessment Arising from Repetitive Actions of the Upper Limbs
Source: Int J Environ Res Public Health. 2020 May 13;17(10):3390. doi: 10.3390/ijerph17103390 (PMC7277380; doi:10.3390/ijerph17103390)
Supplement: Supplementary file 1 [file ijerph-17-03390-s001.pdf]

Zone: Green

Participant	Gender	Age (yeats)	Weight (Kg)	Height (m)	Air temperature (ºC)	Relative humidity (%)	OCRA Index	BMI (Kg/m^2)	Shoulder (ºC)			Forearm (ºC)			Hand (ºC)		
									T0	T20	ΔT	T0	T20	ΔT	T0	T20	ΔT
1	Male	31	76,0	1,77	19,5	53	1,35	24,26	31,2	33,0	**1,8**	29,8	31,9	**2,1**	28,3	28,4	**0,1**
2	Male	25	94,0	1,82	19,4	63	1,45	28,38	31,5	32,0	**0,5**	30,4	30,8	**0,4**	29,8	29,1	**-0,7**
3	Male	35	82,5	1,75	19,7	52	1,41	26,94	33,0	34,3	**1,3**	32,4	33,2	**0,8**	30,4	30,4	**0,0**
4	Male	30	75,0	1,73	21,9	40	1,44	25,06	33,1	33,9	**0,8**	32,3	33,4	**1,1**	32,6	33,7	**1,1**
5	Male	24	107,5	1,94	20,0	54	1,64	28,56	32,0	33,7	**1,7**	30,3	31,8	**1,5**	28,5	30,4	**1,9**
6	Male	25	62,0	1,72	20,0	49	1,45	20,96	31,9	32,4	**0,5**	31,8	32,1	**0,3**	29,9	29,9	**0,0**
7	Male	26	75,0	1,72	20,6	59	1,41	25,35	33,1	34,0	**0,9**	33,7	34,2	**0,5**	34,5	34,7	**0,2**
8	Male	19	59,0	1,72	20,1	62	1,34	19,94	34,1	34,4	**0,3**	32,0	32,3	**0,3**	31,5	30,2	**-1,3**
9	Male	25	79,0	1,79	18,0	44	1,54	24,66	32,7	33,0	**0,3**	30,6	31,5	**0,9**	32,0	30,8	**-1,2**
10	Male	25	90,0	1,79	19,3	41	1,42	28,09	32,5	33,5	**1,0**	31,6	32,2	**0,6**	27,6	27,6	**0,0**
11	Male	38	80,0	1,69	21,8	40	1,31	28,01	32,5	34,1	**1,6**	33,1	34,4	**1,3**	33,6	33,3	**-0,3**
12	Male	23	73,0	1,90	21,0	52	1,36	20,22	33,3	34,7	**1,4**	31,2	32,6	**1,4**	32,7	32,1	**-0,6**
13	Male	21	70,0	1,90	19,1	53	1,37	19,39	33,1	34,0	**0,9**	30,8	30,6	**-0,2**	29,4	28,6	**-0,8**
14	Male	19	54,0	1,68	20,4	52	1,29	19,13	33,5	33,7	**0,2**	31,8	32,6	**0,8**	33,8	30,9	**-2,9**
15	Male	27	60,0	1,78	17,4	57	1,52	18,94	31,8	33,5	**1,7**	30,6	32,2	**1,6**	26,0	27,2	**1,2**
16	Male	29	105,0	1,85	18,7	54	1,44	30,68	31,8	32,8	**1,0**	31,0	31,2	**0,2**	29,0	30,4	**1,4**
17	Female	23	65,0	1,68	18,4	54	1,44	23,03	31,0	32,5	**1,5**	30,4	32,4	**2,0**	26,4	26,3	**-0,1**
18	Female	23	59,0	1,70	20,5	55	1,51	20,42	33,9	34,5	**0,6**	33,3	34,2	**0,9**	33,5	32,5	**-1,0**
19	Female	24	85,0	1,73	22,0	70	1,30	28,40	33,5	33,9	**0,4**	33,7	33,5	**-0,2**	34,1	34,1	**0,0**
20	Female	30	69,0	1,52	20,0	74	1,42	29,86	31,0	31,1	**0,1**	29,6	30,3	**0,7**	30,1	29,4	**-0,7**
21	Female	25	68,0	1,60	18,6	54	1,42	26,56	31,8	32,0	**0,2**	30,9	30,7	**-0,2**	27,3	27,9	**0,6**
22	Female	40	60,0	1,62	19,0	60	1,56	22,86	31,9	31,3	**-0,6**	32,0	31,7	**-0,3**	30,7	29,5	**-1,2**
23	Female	26	62,0	1,70	21,0	54	1,37	21,45	33,9	35,2	**1,3**	31,7	32,8	**1,1**	29,0	30,2	**1,2**
24	Female	26	72,0	1,68	21,6	68	1,38	25,51	33,4	33,9	**0,5**	31,7	32,5	**0,8**	33,6	33,3	**-0,3**
25	Female	25	76,0	1,70	19,2	41	1,29	26,30	32,2	32,6	**0,4**	29,3	29,8	**0,5**	28,1	28,9	**0,8**
26	Female	23	42,0	1,55	21,4	48	1,36	17,48	33,8	34,8	**1,0**	32,1	33,1	**1,0**	32,1	31,4	**-0,7**
27	Female	25	85,0	1,62	20,0	63	1,50	32,39	31,5	31,7	**0,2**	30,8	31,1	**0,3**	32,1	31,0	**-1,1**
28	Female	20	68,0	1,70	19,0	53	1,34	23,53	32,3	33,3	**1,0**	31,5	31,5	**0,0**	29,6	29,8	**0,2**
29	Female	28	60,0	1,76	19,1	67	1,37	19,37	30,2	31,9	**1,7**	29,2	29,3	**0,1**	26,3	25,5	**-0,8**
30	Female	21	52,0	1,65	20,5	48	1,39	19,10	32,4	34,1	**1,7**	31,4	32,2	**0,8**	30,1	30,2	**0,1**
31	Female	23	52,0	1,58	18,7	53	1,38	20,83	31,2	33,0	**1,8**	30,7	32,0	**1,3**	28,4	30,4	**2,0**
32	Female	23	60,0	1,58	18,9	68	1,46	24,03	30,8	31,3	**0,5**	31,0	31,2	**0,2**	32,5	30,6	**-1,9**

Zone: Yellow

Participant	Gender	Age (yeats)	Weight (Kg)	Height (m)	Air temperature (ºC)	Relative humidity (%)	OCRA Index	BMI (Kg/m²)	Shoulder (ºC)			Forearm (ºC)			Hand (ºC)		
									T0	T20	ΔT	T0	T20	ΔT	T0	T20	ΔT
1	Male	31	76,0	1,77	18,8	64	2,09	24,26	30,8	33,2	**2,4**	30,6	32,5	**1,9**	28,8	28,7	**-0,1**
2	Male	25	94,0	1,82	23,1	52	2,13	28,38	33,1	33,7	**0,6**	33,4	33,7	**0,3**	34,6	34,2	**-0,4**
3	Male	35	82,5	1,75	21,8	45	3,37	26,94	33,9	35,1	**1,2**	32,5	34,3	**1,8**	32,1	32,4	**0,3**
4	Male	30	75,0	1,73	20,5	45	3,07	25,06	33,1	34,2	**1,1**	32,7	33,0	**0,3**	33,5	34,0	**0,5**
5	Male	24	107,5	1,94	19,5	49	3,43	28,56	32,7	33,4	**0,7**	31,1	31,5	**0,4**	31,1	31,8	**0,7**
6	Male	25	62,0	1,72	21,4	52	2,03	20,96	32,5	34,0	**1,5**	32,8	33,4	**0,6**	34,6	34,1	**-0,5**
7	Male	26	75,0	1,72	20,9	48	3,03	25,35	31,9	33,8	**1,9**	32,9	33,7	**0,8**	33,8	34,6	**0,8**
8	Male	19	59,0	1,72	20,4	57	1,90	19,94	33,4	34,3	**0,9**	32,2	33,0	**0,8**	28,4	27,5	**-0,9**
9	Male	25	79,0	1,79	19,0	42	3,11	24,66	31,8	32,6	**0,8**	30,6	31,1	**0,5**	32,1	30,4	**-1,7**
10	Male	25	90,0	1,79	21,8	49	2,02	28,09	33,6	34,7	**1,1**	33,7	34,8	**1,1**	34,6	34,8	**0,2**
11	Male	38	80,0	1,69	20,9	43	1,90	28,01	32,2	34,4	**2,2**	32,2	34,1	**1,9**	33,0	32,8	**-0,2**
12	Male	23	73,0	1,90	19,6	54	1,92	20,22	33,4	34,9	**1,5**	32,0	33,2	**1,2**	33,0	32,3	**-0,7**
13	Male	21	70,0	1,90	20,6	60	2,75	19,39	32,5	34,3	**1,8**	30,5	32,0	**1,5**	28,8	29,4	**0,6**
14	Male	19	54,0	1,68	19,1	60	1,98	19,13	33,2	33,9	**0,7**	31,1	33,1	**2,0**	31,5	30,4	**-1,1**
15	Male	27	60,0	1,78	17,4	57	3,02	18,94	32,0	33,8	**1,8**	31,3	32,8	**1,5**	27,2	27,9	**0,7**
16	Male	29	105,0	1,85	21,9	67	2,03	30,68	32,5	33,8	**1,3**	32,9	32,8	**-0,1**	32,8	32,5	**-0,3**
17	Female	23	65,0	1,68	19,2	63	1,90	23,03	32,5	32,5	**0,0**	31,5	32,3	**0,8**	30,1	28,5	**-1,6**
18	Female	23	59,0	1,70	21,1	42	3,01	20,42	34,2	35,0	**0,8**	33,5	34,1	**0,6**	34,3	33,4	**-0,9**
19	Female	24	85,0	1,73	21,6	63	3,03	28,40	32,7	33,2	**0,5**	33,2	33,8	**0,6**	33,9	33,9	**0,0**
20	Female	30	69,0	1,52	21,9	63	3,01	29,86	31,6	32,5	**0,9**	30,8	31,8	**1,0**	30,5	30,1	**-0,4**
21	Female	25	68,0	1,60	20,9	68	2,03	26,56	31,3	32,6	**1,3**	31,9	32,5	**0,6**	32,2	32,5	**0,3**
22	Female	40	60,0	1,62	21,0	54	3,43	22,86	32,8	34,4	**1,6**	32,9	33,4	**0,5**	33,0	32,7	**-0,3**
23	Female	26	62,0	1,70	22,4	46	2,16	21,45	34,0	34,8	**0,8**	31,9	32,1	**0,2**	32,7	31,4	**-1,3**
24	Female	26	72,0	1,68	21,8	63	3,00	25,51	32,6	33,6	**1,0**	30,7	32,1	**1,4**	28,6	29,8	**1,2**
25	Female	25	76,0	1,70	22,2	54	1,95	26,30	33,1	34,4	**1,3**	32,1	32,4	**0,3**	31,3	32,6	**1,3**
26	Female	23	42,0	1,55	20,2	55	2,27	17,48	33,9	34,7	**0,8**	33,7	33,9	**0,2**	30,7	29,7	**-1,0**
27	Female	25	85,0	1,62	20,0	64	2,17	32,39	31,5	32,3	**0,8**	31,4	32,1	**0,7**	33,4	33,3	**-0,1**
28	Female	20	68,0	1,70	21,9	69	2,13	23,53	33,3	35,1	**1,8**	32,2	33,9	**1,7**	32,3	33,4	**1,1**
29	Female	28	60,0	1,76	18,0	48	1,99	19,37	30,4	31,4	**1,0**	29,5	29,8	**0,3**	27,2	26,6	**-0,6**
30	Female	21	52,0	1,65	19,5	52	3,92	19,10	31,8	34,3	**2,5**	30,6	32,1	**1,5**	27,7	29,4	**1,7**
31	Female	23	52,0	1,58	19,8	56	3,12	20,83	33,5	34,1	**0,6**	31,6	32,1	**0,5**	29,1	31,4	**2,3**
32	Female	23	60,0	1,58	19,5	72	3,12	24,03	31,0	31,4	**0,4**	31,2	31,6	**0,4**	33,4	33,1	**-0,3**

Zone: Orange

Participant	Gender	Age (yeats)	Weight (Kg)	Height (m)	Air temperature (ºC)	Relative humidity (%)	OCRA Index	BMI (Kg/m²)	Shoulder (ºC)			Forearm (ºC)			Hand (ºC)		
									T0	T20	ΔT	T0	T20	ΔT	T0	T20	ΔT
1	Male	31	76,0	1,77	23,5	55	4,07	24,26	33,3	34,6	**1,3**	32,5	35,1	**2,6**	31,9	31,7	**-0,2**
2	Male	25	94,0	1,82	23,9	56	3,64	28,38	33,7	35,1	**1,4**	33,1	34,6	**1,5**	34,1	34,1	**0,0**
3	Male	35	82,5	1,75	20,5	50	3,39	26,94	34,2	35,2	**1,0**	32,9	34,6	**1,7**	29,6	30,7	**1,1**
4	Male	30	75,0	1,73	22,0	56	3,89	25,06	32,8	34,6	**1,8**	32,3	33,8	**1,5**	32,6	33,7	**1,1**
5	Male	24	107,5	1,94	20,5	42	3,82	28,56	33,1	34,2	**1,1**	31,7	32,9	**1,2**	30,4	32,0	**1,6**
6	Male	25	62,0	1,72	21,9	45	3,64	20,96	33,1	35,2	**2,1**	32,8	34,2	**1,4**	33,9	33,4	**-0,5**
7	Male	26	75,0	1,72	20,7	57	4,21	25,35	32,5	33,8	**1,3**	33,0	33,8	**0,8**	34,3	34,7	**0,4**
8	Male	19	59,0	1,72	20,7	55	3,25	19,94	33,0	34,5	**1,5**	32,0	33,4	**1,4**	33,4	33,1	**-0,3**
9	Male	25	79,0	1,79	20,8	64	3,69	24,66	32,7	33,8	**1,1**	31,5	33,2	**1,7**	33,4	32,3	**-1,1**
10	Male	25	90,0	1,79	22,3	54	3,51	28,09	34,0	35,2	**1,2**	33,8	35,5	**1,7**	34,2	34,6	**0,4**
11	Male	38	80,0	1,69	21,2	56	3,76	28,01	33,0	34,9	**1,9**	33,6	35,0	**1,4**	34,0	34,2	**0,2**
12	Male	23	73,0	1,90	21,5	54	3,54	20,22	34,5	35,7	**1,2**	32,9	35,0	**2,1**	35,6	34,8	**-0,8**
13	Male	21	70,0	1,90	21,9	68	4,00	19,39	33,0	35,0	**2,0**	31,9	34,1	**2,2**	33,5	33,5	**0,0**
14	Male	19	54,0	1,68	19,2	53	3,58	19,13	33,6	34,9	**1,3**	31,7	33,6	**1,9**	30,1	29,7	**-0,4**
15	Male	27	60,0	1,78	18,0	42	3,53	18,94	32,4	34,1	**1,7**	30,9	33,5	**2,6**	25,2	27,0	**1,8**
16	Male	29	105,0	1,85	19,4	67	3,23	30,68	32,8	33,8	**1,0**	32,0	32,9	**0,9**	32,6	32,3	**-0,3**
17	Female	23	65,0	1,68	23,4	50	3,16	23,03	33,3	34,9	**1,6**	32,1	35,0	**2,9**	30,5	32,6	**2,1**
18	Female	23	59,0	1,70	22,0	59	3,95	20,42	34,1	35,3	**1,2**	32,8	34,2	**1,4**	33,5	33,4	**-0,1**
19	Female	24	85,0	1,73	20,7	58	3,50	28,40	33,1	32,7	**-0,4**	32,5	33,0	**0,5**	33,6	32,8	**-0,8**
20	Female	30	69,0	1,52	23,4	55	4,60	29,86	33,7	34,2	**0,5**	32,5	33,7	**1,2**	34,3	33,8	**-0,5**
21	Female	25	68,0	1,60	20,4	57	3,51	26,56	31,9	33,6	**1,7**	31,4	32,3	**0,9**	29,2	30,7	**1,5**
22	Female	40	60,0	1,62	21,0	43	3,79	22,86	32,9	34,4	**1,5**	33,2	33,4	**0,2**	32,5	32,7	**0,2**
23	Female	26	62,0	1,70	24,2	56	3,73	21,45	34,0	35,9	**1,9**	32,9	34,5	**1,6**	32,6	32,2	**-0,4**
24	Female	26	72,0	1,68	19,3	54	3,53	25,51	30,7	32,9	**2,2**	32,1	33,1	**1,0**	30,8	31,3	**0,5**
25	Female	25	76,0	1,70	22,7	46	3,93	26,30	33,5	34,2	**0,7**	31,9	32,4	**0,5**	32,9	33,1	**0,2**
26	Female	23	42,0	1,55	19,4	51	3,88	17,48	32,8	34,7	**1,9**	33,0	34,2	**1,2**	27,3	28,2	**0,9**
27	Female	25	85,0	1,62	19,4	54	3,83	32,39	31,6	32,3	**0,7**	31,4	32,2	**0,8**	31,2	32,4	**1,2**
28	Female	20	68,0	1,70	20,5	58	3,72	23,53	31,5	34,1	**2,6**	30,5	32,0	**1,5**	31,0	30,3	**-0,7**
29	Female	28	60,0	1,76	20,1	65	3,58	19,37	31,0	32,5	**1,5**	28,9	30,6	**1,7**	26,6	25,6	**-1,0**
30	Female	21	52,0	1,65	18,0	42	3,72	19,10	30,9	33,7	**2,8**	29,6	30,7	**1,1**	25,1	26,4	**1,3**
31	Female	23	52,0	1,58	20,6	48	3,91	20,83	32,2	34,2	**2,0**	32,8	32,8	**0,0**	29,9	32,3	**2,4**
32	Female	23	60,0	1,58	19,5	60	3,81	24,03	31,8	32,1	**0,3**	30,6	32,3	**1,7**	31,4	32,6	**1,2**

Zone: Red

Participant	Gender	Age (yeats)	Weight (Kg)	Height (m)	Air temperature (ºC)	Relative humidity (%)	OCRA Index	BMI (Kg/m²)	Shoulder (ºC)			Forearm (ºC)			Hand (ºC)		
									T0	T20	ΔT	T0	T20	ΔT	T0	T20	ΔT
1	Male	31	76,0	1,77	21,9	48	6,42	24,26	33,4	35,1	**1,7**	32,8	35,3	**2,5**	31,7	31,3	**-0,4**
2	Male	25	94,0	1,82	22,0	42	7,09	28,38	33,6	35,2	**1,6**	33,1	34,7	**1,6**	33,5	33,7	**0,2**
3	Male	35	82,5	1,75	22,7	42	6,40	26,94	33,7	35,9	**2,2**	33,0	35,6	**2,6**	32,0	34,3	**2,3**
4	Male	30	75,0	1,73	23,4	49	7,27	25,06	33,1	33,8	**0,7**	33,2	34,6	**1,4**	33,7	33,3	**-0,4**
5	Male	24	107,5	1,94	20,9	50	6,14	28,56	33,8	34,9	**1,1**	32,0	33,3	**1,3**	32,7	33,6	**0,9**
6	Male	25	62,0	1,72	24,0	55	7,06	20,96	33,9	35,7	**1,8**	34,0	34,8	**0,8**	34,3	34,2	**-0,1**
7	Male	26	75,0	1,72	22,6	55	6,12	25,35	34,3	35,5	**1,2**	34,9	35,1	**0,2**	35,6	35,4	**-0,2**
8	Male	19	59,0	1,72	23,2	57	6,77	19,94	34,2	35,4	**1,2**	33,2	34,3	**1,1**	34,8	34,1	**-0,7**
9	Male	25	79,0	1,79	20,8	40	6,84	24,66	32,7	34,1	**1,4**	31,4	33,4	**2,0**	32,8	32,5	**-0,3**
10	Male	25	90,0	1,79	22,5	63	7,27	28,09	33,7	34,2	**0,5**	33,8	34,8	**1,0**	34,0	32,9	**-1,1**
11	Male	38	80,0	1,69	24,2	56	6,11	28,01	33,6	35,8	**2,2**	33,6	35,7	**2,1**	34,0	35,0	**1,0**
12	Male	23	73,0	1,90	18,8	53	6,21	20,22	32,9	35,0	**2,1**	32,2	33,9	**1,7**	31,5	31,9	**0,4**
13	Male	21	70,0	1,90	20,3	43	5,94	19,39	32,8	35,2	**2,4**	31,5	34,1	**2,6**	29,6	31,8	**2,2**
14	Male	19	54,0	1,68	17,6	56	6,17	19,13	32,8	35,0	**2,2**	30,7	34,1	**3,4**	26,9	29,3	**2,4**
15	Male	27	60,0	1,78	18,0	51	6,24	18,94	32,4	34,3	**1,9**	30,4	34,1	**3,7**	24,7	26,3	**1,6**
16	Male	29	105,0	1,85	20,0	57	5,58	30,68	32,6	33,5	**0,9**	31,9	33,3	**1,4**	33,1	32,0	**-1,1**
17	Female	23	65,0	1,68	21,2	50	6,45	23,03	32,0	34,7	**2,7**	31,1	34,6	**3,5**	28,4	31,2	**2,8**
18	Female	23	59,0	1,70	22,0	55	7,66	20,42	33,2	35,8	**2,6**	33,8	35,2	**1,4**	34,1	34,3	**0,2**
19	Female	24	85,0	1,73	21,9	43	5,83	28,40	32,8	33,1	**0,3**	32,6	34,1	**1,5**	32,4	33,9	**1,5**
20	Female	30	69,0	1,52	21,9	57	6,73	29,86	31,8	33,8	**2,0**	30,8	33,0	**2,2**	30,4	30,8	**0,4**
21	Female	25	68,0	1,60	23,8	55	6,43	26,56	32,5	34,8	**2,3**	32,0	33,6	**1,6**	31,1	33,1	**2,0**
22	Female	40	60,0	1,62	20,7	51	7,15	22,86	32,0	34,9	**2,9**	31,5	34,0	**2,5**	32,3	34,1	**1,8**
23	Female	26	62,0	1,70	23,0	55	6,88	21,45	34,0	36,3	**2,3**	32,5	34,1	**1,6**	31,0	31,2	**0,2**
24	Female	26	72,0	1,68	23,5	50	7,66	25,51	33,3	34,8	**1,5**	32,3	34,8	**2,5**	34,1	33,4	**-0,7**
25	Female	25	76,0	1,70	22,9	55	6,49	26,30	33,7	34,5	**0,8**	32,7	33,2	**0,5**	32,6	32,2	**-0,4**
26	Female	23	42,0	1,55	22,5	47	6,19	17,48	34,1	35,8	**1,7**	33,2	35,6	**2,4**	34,4	34,2	**-0,2**
27	Female	25	85,0	1,62	23,3	46	7,23	32,39	32,8	34,2	**1,4**	31,6	34,1	**2,5**	33,1	32,8	**-0,3**
28	Female	20	68,0	1,70	22,2	54	6,22	23,53	33,5	35,2	**1,7**	31,5	34,8	**3,3**	32,8	33,2	**0,4**
29	Female	28	60,0	1,76	19,9	66	6,27	19,37	30,6	33,4	**2,8**	28,9	30,9	**2,0**	27,2	27,0	**-0,2**
30	Female	21	52,0	1,65	19,2	67	6,42	19,10	31,9	34,4	**2,5**	30,0	32,0	**2,0**	26,8	28,4	**1,6**
31	Female	23	52,0	1,58	19,5	51	6,33	20,83	31,7	33,6	**1,9**	31,8	33,2	**1,4**	30,8	33,0	**2,2**
32	Female	23	60,0	1,58	20,7	50	6,41	24,03	32,5	33,5	**1,0**	31,4	32,5	**1,1**	32,6	33,2	**0,6**

Zone: Purple

Participant	Gender	Age (yeats)	Weight (Kg)	Height (m)	Air temperature (ºC)	Relative humidity (%)	OCRA Index	BMI (Kg/m^2)	Shoulder (ºC)			Forearm (ºC)			Hand (ºC)		
									T0	T20	ΔT	T0	T20	ΔT	T0	T20	ΔT
1	Male	31	76,0	1,77	23,5	55	11,50	24,26	33,4	35,4	**2,0**	31,8	35,7	**3,9**	29,3	33,0	**3,7**
2	Male	25	94,0	1,82	19,7	58	11,43	28,38	31,3	33,8	**2,5**	30,8	33,7	**2,9**	31,4	32,9	**1,5**
3	Male	35	82,5	1,75	22,7	55	13,86	26,94	33,8	36,0	**2,2**	32,3	35,9	**3,6**	30,9	34,5	**3,6**
4	Male	30	75,0	1,73	21,1	50	11,59	25,06	32,6	34,4	**1,8**	31,3	35,4	**4,1**	32,1	33,5	**1,4**
5	Male	24	107,5	1,94	21,5	48	9,65	28,56	33,2	34,4	**1,2**	31,4	33,1	**1,7**	31,2	33,3	**2,1**
6	Male	25	62,0	1,72	22,4	50	9,30	20,96	32,9	35,5	**2,6**	32,2	35,3	**3,1**	32,8	34,0	**1,2**
7	Male	26	75,0	1,72	24,5	37	10,78	25,35	34,6	35,9	**1,3**	34,8	35,8	**1,0**	35,3	35,4	**0,1**
8	Male	19	59,0	1,72	21,4	70	10,19	19,94	32,3	34,1	**1,8**	31,9	34,0	**2,1**	33,1	32,2	**-0,9**
9	Male	25	79,0	1,79	22,9	57	7,98	24,66	33,1	35,2	**2,1**	31,6	34,9	**3,3**	33,8	33,6	**-0,2**
10	Male	25	90,0	1,79	23,4	60	10,10	28,09	34,0	35,8	**1,8**	32,7	35,4	**2,7**	32,8	34,0	**1,2**
11	Male	38	80,0	1,69	20,0	48	9,22	28,01	32,2	35,2	**3,0**	32,2	35,4	**3,2**	32,9	34,6	**1,7**
12	Male	23	73,0	1,90	21,8	47	10,04	20,22	33,7	35,7	**2,0**	32,0	35,1	**3,1**	32,4	32,7	**0,3**
13	Male	21	70,0	1,90	21,6	54	9,49	19,39	33,9	35,4	**1,5**	31,8	34,5	**2,7**	30,6	32,9	**2,3**
14	Male	19	54,0	1,68	21,5	70	9,06	19,13	34,6	36,0	**1,4**	33,2	35,5	**2,3**	32,3	34,4	**2,1**
15	Male	27	60,0	1,78	21,0	70	9,61	18,94	33,0	35,4	**2,4**	32,0	35,7	**3,7**	29,8	31,5	**1,7**
16	Male	29	105,0	1,85	19,1	54	9,11	30,68	31,8	32,4	**0,6**	30,7	34,0	**3,3**	28,6	29,2	**0,6**
17	Female	23	65,0	1,68	21,5	55	11,21	23,03	32,8	34,6	**1,8**	32,9	34,7	**1,8**	29,7	32,1	**2,4**
18	Female	23	59,0	1,70	21,9	48	10,32	20,42	33,0	35,1	**2,1**	34,2	35,1	**0,9**	31,9	32,9	**1,0**
19	Female	24	85,0	1,73	22,4	55	9,72	28,40	33,3	34,7	**1,4**	32,8	34,7	**1,9**	33,9	34,8	**0,9**
20	Female	30	69,0	1,52	21,4	50	10,20	29,86	32,7	33,5	**0,8**	30,2	33,2	**3,0**	30,7	30,8	**0,1**
21	Female	25	68,0	1,60	22,4	52	10,94	26,56	32,4	34,5	**2,1**	31,3	33,9	**2,6**	31,1	33,5	**2,4**
22	Female	40	60,0	1,62	23,5	44	11,40	22,86	33,5	36,6	**3,1**	32,4	35,4	**3,0**	32,8	34,8	**2,0**
23	Female	26	62,0	1,70	23,4	35	8,79	21,45	34,6	35,5	**0,9**	32,3	33,9	**1,6**	33,7	32,3	**-1,4**
24	Female	26	72,0	1,68	23,2	55	11,52	25,51	33,7	35,1	**1,4**	33,0	35,4	**2,4**	33,3	33,5	**0,2**
25	Female	25	76,0	1,70	23,2	57	11,74	26,30	32,8	33,4	**0,6**	31,2	32,5	**1,3**	31,4	32,0	**0,6**
26	Female	23	42,0	1,55	23,0	57	8,84	17,48	33,5	34,9	**1,4**	33,4	35,1	**1,7**	34,4	33,6	**-0,8**
27	Female	25	85,0	1,62	24,2	57	9,83	32,39	33,4	34,2	**0,8**	33,0	34,4	**1,4**	33,5	33,3	**-0,2**
28	Female	20	68,0	1,70	21,0	52	9,97	23,53	32,6	34,5	**1,9**	30,5	33,7	**3,2**	31,2	32,7	**1,5**
29	Female	28	60,0	1,76	19,0	55	10,50	19,37	31,2	34,0	**2,8**	29,8	32,4	**2,6**	26,8	28,5	**1,7**
30	Female	21	52,0	1,65	21,8	70	10,26	19,10	33,3	35,0	**1,7**	32,0	33,5	**1,5**	32,5	32,2	**-0,3**
31	Female	23	52,0	1,58	20,5	47	9,71	20,83	32,4	34,8	**2,4**	31,7	33,4	**1,7**	30,4	33,4	**3,0**
32	Female	23	60,0	1,58	20,9	52	10,48	24,03	32,0	33,3	**1,3**	31,1	33,4	**2,3**	32,4	33,7	**1,3**
